# Transcending the Gender Binary under International Law: Advancing Health-Related Human Rights for Trans* Populations

**DOI:** 10.1017/jme.2022.84

**Published:** 2022

**Authors:** Aoife M. O’Connor, Maximillian Seunik, Blas Radi, Liberty Matthyse, Lance Gable, Hanna E. Huffstetler, Benjamin Mason Meier

**Affiliations:** 1: UNC HEALTH & HUMAN RIGHTS WORKING GROUP, CHAPEL HILL, NC, USA; 2: UNIVERSITY OF BUENOS AIRES, BUENOS AIRES, ARGENTINA; 3: GENDER DYNAMIX, CAPE TOWN, SOUTH AFRICA; 4: WAYNE STATE UNIVERSITY, DETROIT, MI, USA; 5: THE UNIVERSITY OF NORTH CAROLINA, CHAPEL HILL, NC, USA.

**Keywords:** Transgender, Gender-Diverse, Gender Binary, Human Rights, Right to Health

## Abstract

Despite a recent wave in global recognition of the rights of transgender and gender-diverse populations, referred to in this text by the umbrella label of trans*, international law continues to presume a cisgender binary definition of gender — dismissing the lived realities of trans* individuals throughout the world. This gap in international legal recognition and protection has fundamental implications for health, where trans* persons have been and continue to be subjected to widespread discrimination in health care, longstanding neglect of health needs, and significant violations of bodily autonomy.

## Introduction

Throughout recorded history, and across every country, culture, and region around the world, characteristics of gender and bodily diversity have existed outside the structure of the dominant cisgender male/female gender binary that is recognized today under international law. The emergence and dominance of this binary is intimately tied to violent colonial practices of oppression and capitalism that flatten the complexity of traditions that were once more open to gender diversity.^1^ Despite its comparatively modern invention, the gender binary upheld today has been used by international institutions as a foundational ordering principle, classifying human beings into two socially and biologically distinct categories: male assigned persons who are expected to identify as boys and men and perform masculinity; and female assigned persons who are expected to identify as girls and women and perform femininity. In present discourse, terms such as “transgender,” “gender-diverse,” and the umbrella label of “trans*”^2^ have come to be used on the global stage to refer to those with different gender identities and expressions that do not adhere with the binary cisgender prescript.^3^ Recently, many global institutions have come to acknowledge gender diversity, and specifically gender identity — an individual’s deeply personal experience of gender that may or may not correspond with their sex assigned at birth — as integral to individual dignity and common humanity. However, despite growing international support for protections of gender identity alongside sexual orientation, the human rights community has been slow to recognize the equal dignity and needs of trans* individuals. The resulting gaps in legal protections have rendered these communities at risk of widespread violence, health neglect, and pernicious discrimination. Although the United Nations (UN) human rights system has taken steps in recent decades to strengthen the rights of trans* people, significant violations of human rights continue to occur — both under color of law and because of the international legal system’s inability to address the multiple and intersecting oppressions faced by trans* persons in their lived realities — posing dire threats to the achievement of the highest attainable standard of health for the community.

With significant work ahead to address the egregious health disparities experienced by the trans* community, this article examines how legal recognition of gender identity and expression under international human rights law is needed to advance global health and human rights.

Despite institutional mandates across UN institutions to protect the fundamental right to health of all human beings, spanning every facet of their lived experience, trans* individuals face unique and substantial barriers to health care and underlying determinants of health.^4^ Central to this widespread inequity, the diverse gender identities and gender expressions present within trans* communities have long been pathologized as a mental health disorder — perceived as a condition to be cured. Although advocates both within and outside the health sector have increasingly sought to depathologize trans* identities and secure access to gender-affirming care, legal obstacles persist in much of the world. Such obstacles have exacerbated stigma and discrimination toward trans* persons and undermined the foundational notion that all people are equal in dignity and rights. Without legal recognition of gender identity, trans* individuals face a greater risk of violations of their right to health and bodily autonomy. Governments and medical institutions across the globe have continued to impose forced, coercive, and medically unnecessary procedures on trans* populations, such as mandating sterilization as a pre-condition of changing one’s gender marker, a policy that is now recognized by the UN as a form of torture.^5^ Discrimination, both within and beyond healthcare, is encoded in legislation and perpetuated in policy and practice across nations. As a result, trans* individuals endure glaring health disparities, including staggering rates of domestic and sexual violence, poor mental health outcomes, shortened life expectancy, as well as abuse and neglect at the hands of health workers and service providers. Faced with conditional recognition, or the complete absence of legal recognition, associated rights protections, and social supports, trans* communities face injustice at every turn. Compounded by misogyny, racism, xenophobia, and stigma — these violations affect housing, education, employment, and public accommodations for trans* persons. This ever-present stigmatization, oppression, and discrimination exacerbates the health inequities experienced by trans* communities, contributing to poor health outcomes and revealing gaps in how international legal protections have failed to protect persons with diverse gender identities and expressions.

With significant work ahead to address the egregious health disparities experienced by the trans* community, this article examines how legal recognition of gender identity and expression under international human rights law is needed to advance global health and human rights. The article begins by chronicling the long evolution of international law to recognize the rights of trans* populations, detailing how the UN’s international legal mechanisms have slowly shifted from a rigid gender binary to advance health-related human rights for trans* communities. Yet, this political landscape within the UN has been upended by a growing “anti-gender” backlash, brought forth by movements that vehemently reject the notion of gender as a socio-cultural construct and that view the significant global progress made to advance recognition for trans* persons as a threat to the “traditional” patriarchal, cisnormative, and heteronormative family model. Building upon the growing legal recognition of trans* populations amidst this contested international political environment, this analysis then examines the ways in which health-related human rights can shape trans* health — under attributes of the right to health and principles of the rights-based approach to health. The article concludes by refuting the faulty claims of anti-gender movements and analyzing how the incorporation of a more inclusive gender-expansive framework under international human rights law could strengthen health-related rights for trans* persons, cisgender women and girls, and other marginalized communities.

## I. International Law Presumes a Gender Binary

The evolution of international law has sought since the establishment of the UN to protect women’s rights as human rights, yet it has done so in ways that reify a gender binary, addressing discrimination on the basis of “sex” while neglecting gender identity, sexuality, and bodily diversity under human rights law.

### A. Protecting Women’s Rights

Adopted in 1945, the Charter of the United Nations outlines a goal “to reaffirm faith in fundamental human rights, in the dignity and worth of the human person, [and] in the equal rights of men and women.”^6^ From this architecture for human rights promotion in UN governance, women delegates came together in 1946 to form an independent UN Commission on the Status of Women (CSW), working within the UN to ensure the adequate protection of women’s rights and assure equal rights between gender conforming cisgender men and women. These delegates pushed the UN Human Rights Commission to apply human rights to “all human beings” — rather than its original language, “all men” — in the 1948 Universal Declaration of Human Rights (UDHR). In its final form, the UDHR sought to uphold the rights of all human beings without discrimination, recognizing that: “Everyone is entitled to all the rights and freedoms set forth in this Declaration, without distinction of any kind, such as race, colour, sex, language, religion, political, or other opinion, national or social origin, property, birth or other status.”^7^

Extending the protection of women’s rights, the UN General Assembly adopted the 1967 Declaration on the Elimination of Discrimination Against Women,^8^ which laid a foundation for the codification of human rights in the 1979 Convention on the Elimination of All Forms of Discrimination against Women (CEDAW). This new human rights treaty required states to take “all appropriate measures, including legislation, to ensure the full development and advancement of women, for the purpose of guaranteeing them the exercise and enjoyment of human rights and fundamental freedoms on a basis of equality with men.”^9^ In doing so, CEDAW affirmed that women’s health and reproductive rights are central to women’s equality, prohibiting discrimination against women based upon their role in “procreation” and “maternity,” but situating women’s reproductive and social roles within the context of marriage between a man and a woman. During this time, international human rights law increasingly framed rights under a binary definition of gender — reducing body types to two genders based on biological sex through the “forced unity of sex and gender.”^10^ In this context, emerging second wave feminists were seen to be hostile to the concerns of the trans* community, despite growing legal and social victories emerging in the 1970s for the trans* community around the world.^11^ Because of this ideological state of affairs, the development of international law during this time reflected advocacy that worked to ensure equality between “two sexes,” men and women, based on a gender binary — in and of itself a social construct — that regulates and controls bodies.^12^

### B. Neglecting Sexuality

The end of the Cold War provided new opportunities to restate human rights for a new era, but efforts to extend women’s sexual and reproductive health and rights (SRHR) neglected to encompass issues of sexuality. Women’s rights advocates sought expansive goals to advance women’s health, reproduction, and sexuality as matters of human rights.^13^ Through the 1993 World Conference on Human Rights, states reaffirmed in the resulting Vienna Declaration and Programme of Action that “the human rights of women and of the girl child are an inalienable, integral and indivisible part of universal human rights,” recognizing “the importance of the enjoyment by women of the highest standard of physical and mental health throughout their life span.”^14^ As advocates pressed the SRHR agenda forward, the 1994 International Conference on Population and Development (ICPD) in Cairo shifted toward a rights-based approach that valued women and girls’ rights to choice in reproduction, recognizing “the basic right of all couples and individuals to decide freely and responsibly the number, spacing, and timing of their children.”^15^ This human rights agenda was advanced further in 1995, with the World Conference on Women in Beijing affirming that “the human rights of women include their right to have control over and decide freely and responsibly on matters related to their sexuality, including sexual and reproductive health, free of coercion, discrimination and violence.”^16^

Although the Cairo and Beijing conferences delivered most of the elements of the “Women’s Platform,” they were unable to secure recognition on “sexual rights,” which included two separate but interlinked issues: protections on the basis of sexual orientation and gender identity, and the rights of women and girls to have control over their bodies and sexualities.^17^ Sexual rights faced wide-ranging challenges at the Cairo and Beijing conferences. Although lesbian activists sought the inclusion of sexual orientation in the Beijing program, protesting the assumption of heteronormativity within the existing women’s right framework, each proposal to include language on sexual rights and sexual orientation faced intense backlash.^18^ This political backlash, from an alliance between the Holy See and other conservative nations, sought the preservation of the “traditional family” and promotion of the role of women as wives and mothers. Thus, despite success in recognizing women’s autonomy over sexuality, the term “sexual rights” and all four references to sexual orientation were removed. Where advocates lamented the absence of sexual rights coming out of Cairo and Beijing, with an expanding HIV/AIDS pandemic revealing the perils of neglecting sexuality under international human rights law, the rise of lesbian, gay, bisexual, transgender, queer, and intersex (LGBTQI+) advocacy at the international level was seeking to advance human rights related to sexual orientation and gender identity (SOGI) (as well as rights pertaining to sex characteristics (SOGIESC) — to address unique violations of the rights of intersex persons).

## II. The Evolution of SOGI Rights

As the growing HIV/AIDS crisis demonstrated the urgency in recognizing sexuality and strengthening protections of LGBTQI+ persons under international human rights law, the early SOGI rights movement was making a powerful entrance onto the multilateral stage. From the riots at Stonewall to the halls of the UN, over 1,000 LGBTQI+ persons marched in 1984 to the UN headquarters in New York, demanding an end to the pathologization of LGBTQI+ identities, discriminatory laws and policies, and structural violence faced by the community. As early legal recognitions for the LGBTQI+ community advanced outside of UN bodies, steady pressure from community advocates led in 2011 to the adoption of the first UN General Assembly resolution that addressed human rights violations based on both sexual orientation and gender identity. However, this advancement was not without contention from both within and outside of the LGBTQI+ and feminist movements. While these advancements reflected a united advocacy front among LGBTQI+ persons, under the surface lay ongoing divisions between LGBTQI+ groups — based not only on sexual identity and gender expression, but also across lines of race, class, geography, and other intersecting identities, which have influenced the ways in which the rights of trans* communities have been conceptualized and advanced.

### A. The Early SOGI Rights Movement

Concurrent with human rights advances across global human rights governance, the 1990s saw rising global support for the depathologization and destigmatization of trans* identities, challenging the dominant narrative of a supposed natural binary gender order.^19^ Leading up to the 1990s, this movement was energized by LGBTQI+ advocates, who first achieved preliminary progress toward depathologization in 1987, when the U.S. Diagnostic and Statistical Manual of Mental Disorders III (DSM-III) removed reference to “ego-dystonic homosexuality” — a clinical diagnosis that legitimized harmful sexual orientation change efforts, such as conversion therapy. Although this reference was replaced in the DSM-III-R with a more generalized mention of “persistent and marked distress about one’s sexual orientation,” the moment marked an important step toward the full removal of homosexuality within the DSM, a milestone that was later achieved in 2013 through the publication of the DSM-5.^20^ This gradual removal of stigmatizing, pathological framings of homosexuality in the DSM was followed by the World Health Organization’s (WHO) International Classification of Diseases (ICD), eliminating “ego-dystonic homosexuality” in 2014 within the published draft of ICD-11 (formally going into effect in January 2022).^21^ From these advances for gay and lesbian communities, demands from international activists grew regarding the depathologization of trans* identities, gaining support from international bodies, including the Council of Europe and the European Parliament.^22^ While well-intentioned, many of the actions taken to advance depathologization efforts for trans* populations have fallen short, and some efforts have been complicated or even proved counterproductive. For example, while the 1979 formation of the World Professional Association for Transgender Health (WPATH) — one of the first organizations to combine international trans* advocacy with recognition of the human right to health — has been lauded by many, the organization has also been widely critiqued. This critique centers around WPATH’s history of upholding the expertise of predominantly cisgender clinicians, as well as the role it plays in the continued pathologization of trans* people through its widely-used Standards of Care, which requires trans* clients to access a mental health evaluation and meet the diagnostic criteria for “gender dysphoria” before gaining access to hormonal or surgical interventions^23^ — as opposed to alternative models of care based on gender self-determination, such as the Informed Consent Model of Transgender Care.^24^ Yet even as the pathologization of trans* identities continues in many medical contexts today, this stance has faced strong international condemnation from human rights advocates, including in 2017 by the UN Special Rapporteur on the right to health, who denounced its reduction of the identities of transgender and intersex persons to diseases in ways that have exacerbated global stigma and discrimination.^25^

As these conversations across the medical and legal communities continued to play out across national and international forums, growing movements for trans* rights began appearing around the world. Even in regions traditionally hostile to the rights of LGB identified individuals, pockets of progress regarding legal recognition for trans* individuals were beginning to emerge, with examples from conservative states like Iran and Egypt, where sex-reassignment surgery was legalized in the 1980s by the fatwas of Ayatollah Khomeini in Iran and Sheikh Muhammad al-Tantawi in Egypt.^26^ In many regions around the world, growing recognition was strengthened by the formation of new national and international advocacy organizations, such as Red Latinoamericano y del Caribe de Personas Trans (2004), Transgender Europe (2005), Gender Dynamix (2006), Pacific Sexual Diversity Network (2007), Asia Pacific Transgender Network (2008), and Global Action for Trans Equality (2010).^27^ Despite a lack of significant legal recognition in UN bodies, legal claims began appearing in regional courts around the world, leading to ground-breaking jurisprudence that recognized trans* rights.^28^

Coinciding with this push by NGOs and the LGBTQI+ movement to gain wider acceptance and legal protections for sexual orientation and gender identity,^29^ links between human rights and SOGI began to surface in the international agenda among special and thematic UN forums^30^ and within the language of General Assembly resolutions^31^ throughout the 1990s and the early 2000s. Although these advancements marked a notable shift in the direction of progress for the advocacy community within multilateral fora, especially given the contentious nature of SOGI rights at the turn of the century, UN Special Procedures mandate holders — individual representatives rather than state delegates — played an essential early role in both raising and clarifying SOGI issues throughout this period.^32^

As the SOGI movement’s influence continued to grow, the term “sexual rights” — which had failed to secure recognition at the UN during the Cairo and Beijing conferences — was further legitimized within the human rights community following its 2004 use by the UN Special Rapporteur on the right to health. Considering the SRHR provisions advanced in Cairo and Beijing, the Special Rapporteur noted that “since many expressions of sexuality are non-reproductive, it is misguided to subsume sexual rights, including the right to sexual health, under reproductive rights and reproductive health.” Sexual rights, the Special Rapporteur concluded, include the “right of all persons to express their sexual orientation, with due regard for the well-being and rights of others, without fear of persecution, denial of liberty or social interference.”^33^

Feminist and LGBTQI+ movements increasingly came to recognize sexual rights as separate and distinct from reproductive rights, with advocates pushing for the inclusion of specific language on both sexual rights and sexual orientation across UN working groups. However, while sexual rights gained significant traction, distinct rights related to gender identity and bodily diversity would continue to remain unacknowledged in multilateral fora until 2006. This is a standing reflection of the persistence of the male/female gender binary presumed under international law, and concurrently influenced by social hierarchies within the LGBTQI+ movement, which privileged those that most closely fit within the binary.^34^ In particular, there was tension around the political viability of advocating for trans* rights as compared to lesbian, gay, and bisexual rights, the latter of whose identities more neatly conformed with the societal expectations of the Western middle class (i.e. relationships between two cis-gendered people).^35^ Yet for many opponents of the LGBTQI+ movement, anti-gay and anti-trans* sentiments were one in the same. Given this shared opposition, trans* leaders moved to have trans* rights incorporated into the agendas of established gay and lesbian advocacy organizations.^36^ Amid this disjointed progress seen within the “SO/GI community,” a larger global reckoning surrounding the interpretation of “gender” was on the horizon for feminists, LGB activists, and conservative member states. Across the international stage, the term ‘gender’ came to represent two critical pathways of legal interpretations: (1) a primary focus on combatting discrimination and disparities against cisgender women and girls and (2) a newer interpretation pertaining to gender identity and expression, with the latter calling into question the use of the gender binary under the international legal framework.^37^

As these conversations continued to unfold, advocates working across the full spectrum of SOGI rights achieved a breakthrough in 2006 through a ground-breaking set of principles on SOGI rights and international law, the Yogyakarta Principles. Developed in Indonesia by a group of international legal experts, the Yogyakarta Principles define state obligations toward LGBTQI+ populations in relation to an expansive range of civil, political, economic, and social rights. In response to well-documented patterns of SOGI-based violence, discrimination, and abuse, the principles name a number of abuses and areas of concern — including extra-judicial killings, torture, and sexual assault due to someone’s perceived or actual sexual orientation or gender identity — and further provide detailed recommendations on how states may better protect the human rights of LGBTQI+ people across the world.^38^ Advancing efforts to depathologize and protect trans* populations from medical abuse, the Yogyakarta Principles make clear:

No person may be forced to undergo any form of medical or psychological treatment, procedure, testing, or be confined to a medical facility, based on sexual orientation or gender identity. Notwithstanding any classifications to the contrary, a person’s sexual orientation and gender identity are not, in and of themselves, medical conditions and are not to be treated, cured, or suppressed.^39^

The Yogyakarta Principles achieved a high level of visibility following their public launch, gaining immediate support from human rights NGOs, state governments across Europe and the Americas, and in convenings across regional human rights organizations and legal forums. Through these foundational Principles, advocates across the SOGI movement took a giant step forward in gaining global recognition for the applicability of international legal standards to address the rights of lesbian, gay, bisexual, and trans* individuals around the world.^40^

### B. SOGI Resolutions and Yogyakarta Principles Plus 10

Despite the widespread influence of the 2006 Yogyakarta Principles, the first formal UN resolution on SOGI rights would not be adopted for another five years. Issues related to sexual orientation and gender identity remained highly contested among multilateral fora, with the causes of resistance ranging from “religiosity, a lack of modernity, a counter-reaction to Western LGBTQI+ activism, and states scapegoating LGBTQI+ persons for political goals such as nation-building, diverting attention from other problems, shoring up authority, or to use as an international bargaining chip.”^41^ As such, the adoption of UN resolutions to protect SOGI-based rights proved slow and difficult, with states unwilling to lead on the issue.

While the UN General Assembly has repeatedly called attention to rights violations of persons based on sexual orientation since 2003 — notably through its resolutions on extrajudicial, summary, and arbitrary killings — rights violations based on gender identity would not be recognized until much later.^42^ The UN Human Rights Council (HRC) brought its first successful SOGI resolution to the table in June 2011 — to request an investigative study by the UN High Commissioner on Human Rights to examine how international human rights law could be used to end human rights violations on the basis of both sexual orientation and gender identity. Despite grave concern over high rates of SOGI-based violence and discrimination reported around the world, the resolution was only narrowly adopted (23 members in favor, 19 against, and 3 abstentions), seeking a finalized report on SOGI discrimination by December 2011.^43^ The final report of the High Commissioner, while developed rapidly over only a few months, would be celebrated by LGBTQI+ advocates, who recognized that “the United Nations had unequivocally affirmed that the protections guaranteed by the Universal Declaration of Human Rights apply to each and every one of us.”^44^

Following this first resolution and report from the HRC, a 2014 follow-up resolution welcomed positive developments but reiterated the Council’s concern over SOGI-based violence and discrimination. While the HRC vote again remained narrow (with 25 in favor, 14 against, and 7 abstentions), there was a marginal increase in member support since the first action was taken three years earlier.^45^ With support for SOGI rights slowly building among states, the HRC in 2016 appointed the first Independent Expert to identify ways to “assess the implementation of existing international human rights instruments with regard to ways to overcome [SOGI-based violence and discrimination].”^46^

In keeping with the normative evolution of international human rights law, and following both the appointment of the SOGI Independent Expert and the adoption of the 2006 Yogyakarta Principles, a group of experts convened in Geneva in 2017 to integrate the significant developments that had been made in the UN to understand and recognize SOGI-related rights violations — adopting an additional set of principles and state recommendations, the “Yogyakarta Principles plus 10.” These updated Principles not only reflected a more detailed understanding of the violations affecting persons of diverse sexual orientations, gender identities, and sex characteristics, including trans* and intersex persons, but further recognized the distinction between gender expression, sexual orientation, and sex characteristics — and the distinct rights violations that occur on the grounds of each.^47^ With this new contribution to the advancement of international human rights law, the Yogyakarta Principles plus 10 helped chart the way forward for the future of SOGIESC-rights on the international legal stage.

### C. “Anti-Gender” Backlash at the UN

While significant global progress has been made in recent decades, culminating in the Yogyakarta Principles plus 10, these advancements in international recognition of trans* rights have not moved forward without substantial struggle. Opposition to the advancement of trans* rights has often been framed in UN debates as a resistance to the imposition of what SOGI-rights opponents have termed “gender-ideology” — encompassing a purported attempt by Western governments to “attack” traditional notions of family, culture, and national identities.^48^ This opposition to trans* rights and SOGI movements has typically been brought forth by those with a background of religious fundamentalism or conservative nationalism (from states that enforce rigid interpretations and expectations of societal identities and behaviors), but such opposition has also arisen from within mainstream feminist and LGB movements.^49^

Early opposition to the SOGI movement in UN debates can be traced back to the Cairo conference, where conservative UN member states such as the Holy See, Libya, and Iran found common purpose in the corresponding antifeminist movement and worked to resist the legal recognition of women’s reproductive autonomy.^50^ This early development of an anti-feminist and anti-gender movement at the UN, heralding the preservation of traditional family values, has grown over time to stymie a myriad of related SRHR issues, including comprehensive sexuality education, sex workers’ rights, protections around abortion, as well as LGBTQI+ protections and provisions in international law.^51^ In recent years, however, this traditionally conservative anti-gender movement has found unlikely allies from within the LGB and feminist movements.^52^ Exploiting tensions across feminist groups, recent human rights debates have seen the rise of a newly emboldened global ideology of anti-trans feminism, using the term “gender-critical” to define themselves — and defined in broader circles as “Trans Exclusionary Radical Feminists” (TERFs).^53^ Although the ideology and activism behind the TERF movement can be traced back to the United States in the 1960s, this ideology has since found its way across the Atlantic, with the U.S. based TERF organization, Women’s Declaration International (WDI), receiving signatories from individuals in over 150+ countries, and from over 400 organizations for its “Declaration on Women’s Sex-Based Rights.”^54^ Despite its origins as a fringe political movement, the TERF movement has found a strong foothold in both mainstream U.S. and U.K. politics, rivaling the political power of traditional conservative forces due to their ability to market themselves as a centrist ideology that bridges both the feminist left and the conservative right.^55^ Through the alignment of both the TERF movement and conservative forces, this growing anti-trans* movement, grounded in a gender essentialism that posits biological sex as the determining status for gender, has found political support for the argument that trans* rights and a more fluid and expansive understanding of gender identity are a threat to the hard-fought human rights of cisgender women and girls.^56^

With growing alignment among anti-trans* forces — including the international TERF movement, and more traditional anti-SOGI actors such as conservative UN member states and prominent conservative NGOs — the advancement of trans* rights has faced substantial backlash. In reaction to the initial landslide of progress made by SOGI activists during the paradigm shift of the early 2000s, this collective backlash against the advancement of human rights for trans* populations has slowed progress for trans* communities in regional and national courtrooms — targeting rights around access to gender-affirming legal documentation, access to safe bathroom facilities, and numerous other issues that underlie health and wellbeing.^57^ With growing political and monetary strength, the international anti-gender movement has been found to have received $3.7 billion worldwide between 2013 and 2017 — while LGBTQI+ movements had received only $1.2 billion for the advancement of SOGI rights.^58^

By marketing the advancement of SOGI-rights as an alleged threat to women, children, and families, the anti-gender movement has worked in the UN to position support for SOGI rights as an oppositional stance to the important achievements made thus far on gender equality and the protection of women, girls, and other minority communities from discrimination. In response, SOGIESC rights advocates have taken intentional action to highlight how the work to eradicate discrimination on the basis of gender identity operates harmoniously with the continued movement to advance the rights of women and girls around the world, noting the importance of synergistic movements to eradicate gender-based discrimination for all human beings, regardless of their gender identity, expression, or sex characteristics.^59^ As international human rights law faces an important crossroads in the UN, these interpretations of gender under international human rights law will have crucial implications for the health-related human rights and well-being of trans* populations.

## III. Conceptualization of Health-Related Trans* Rights

Human rights norms and principles align with and support the realization of health-related trans* rights, framing health policies, programs, and practices that impact trans* persons. Grounded in human rights under international law, the foundational assertion that “all humans are born free and equal in dignity and rights” has provided a means to realize the universal freedoms and entitlements that underpin the health of trans* individuals. Given the range of obstacles trans* persons must overcome to achieve health, the norms of the human right to health and the principles of the rights-based approach to health offer a roadmap to realize gender self-determination, freedom of expression, bodily autonomy, respect, and an expansive vision of health around which advocates, policymakers, trans* patients, and communities can come together. The foundation of human rights in universal equality provides a starting point from which health care and underlying determinants of health can be assessed — applying a wider range of health-related human rights to the development of health policy. Such health-related human rights offer a common moral language with associated legal obligations from which rising demands to improve the health of trans* people can be translated into changes in policies, programs, and practices — guided by the right to health and rights-based approach to health.

### A. The Right to Health and Trans* Health

The right to health plays a crucial role in framing government efforts to progressively realize health care and underlying determinants of health for trans* populations. In elaborating the normative content of the right to health, the UN Committee on Economic, Social and Cultural Rights (CESCR) has identified specific freedoms and entitlements that apply to all individuals. Under General Comment 14 (adopted in 2000) to the International Covenant on Economic, Social and Cultural Rights (ICESCR), the CESCR articulated a framework for the normative content of the right to health, affirming each individual’s agency to make decisions about their own health and their right to not be subjected to harmful practices that undermine health. Among the freedoms outlined in this General Comment, the CESCR delineated “the right to control one’s health and body, including sexual and reproductive freedom, and the right to be free from interference, such as the right to be free from torture, non-consensual medical treatment and experimentation.”^60^ In realizing the right to health for trans* populations, General Comment 14 specifies key attributes of the right to health, assessing health determinants on the basis of their availability, accessibility, acceptability, and quality.

To satisfy the availability of the right to health, governments should ensure a sufficient quantity of “functioning public health and health care facilities, goods and services, as well as programs.”^61^ In the context of trans* health, availability requires the state to progressively ensure the types of health care services provided meet the needs of trans* people. This includes ensuring health care providers and public health experts understand the unique health needs of trans* people and that service provision adapts to changes in best practices. Situating trans* health within inclusive and comprehensive national health policies for universal health coverage can bolster attention and help muster the resources necessary to provide gender-affirming health care and public health services.

The human rights attribute of accessibility obliges states to reduce and remove physical, economic, geographic, and informational obstacles to health care and public health — barriers that have frequently undermined access to gender-affirming care for trans* populations.^62^ While circumstances vary considerably across and within national contexts, the cost of health care, including access to feminizing and masculinizing hormones, gender-affirming surgeries (e.g. chest reconstruction surgery, vaginoplasty, phalloplasty, metoidioplasty, etc.), puberty blockers, mental health supports, and other “invisible costs,” is frequently inaccessible and/or unaffordable, preventing trans* individuals from receiving necessary medical care.^63^ Accurate information about effective clinical service provision and the epidemiology of communicable and non-communicable diseases in trans* people globally is also lacking.^64^ Additionally, representations of trans* health issues in both literature and practice are too often relegated to a narrow dimension of sexual health focusing primarily on the disproportionate HIV and STI burden shouldered by this population. This demands an important shift in the global conversation towards embracing trans* health as a comprehensive state of complete physical, mental, and social well-being.^65^ These information gaps place intrinsic constraints on the accessibility of trans*-specific care, especially when it comes to information essential to improving knowledge and care for different subgroups within the trans* community through an intersectional human rights lens. This may include transgender men, those who also identify as non-binary or intersex, the elderly, and those residing in rural, remote, or hard to reach places.^66^ Compounded by discrimination, limitations in accessibility to both health care itself and the underlying determinants of health, presents what has been described as a “slope leading from stigma to sickness.”^67^ Implementing rights-protective laws and social policies to curtail discrimination, violence, and stigma against trans* people can foster greater accessibility of health care and public health services, as exemplified by Argentina — often seen as a pioneer regarding trans* inclusive policies — who passed legislation in 2012 upholding the right of all citizens above the age of 18 to access free surgical and comprehensive hormonal interventions without court authorization, as well as the ability to obtain a government issued I.D. with their chosen gender, without the need to undergo any medical or psychological gatekeeping.^68^ Beyond prohibiting discriminatory barriers to access, states seeking to uphold the right to health should: implement publicly funded programs that cover the costs of necessary medical care for trans* persons; expand investment into the provision of clinical care across a wide geographic area; and provide accessible information to improve care for and understanding of diverse trans* communities.

Even where health services are available and accessible to trans* people, many providers are insufficiently trained to provide acceptable care to trans* patients. Individual medical interventions may fail to appreciate the overarching structural context, local realities, and cultural and religious dynamics that trans* patients experience, limiting their effectiveness.^69^ Trans* patients may also avoid institutions or healthcare providers that require disclosure of their gender history in an effort to protect themselves from pervasive stigma.^70^ In some settings, trans* patients may need to endure onerous and arbitrary legal and administrative gender recognition processes to become eligible for desired medication and surgical procedures. In the process, trans* persons may be forced to undergo invasive, unnecessary, and unethical medical interventions to satisfy various preconditions to gender recognition.^71^ Further, when trans* people present with gender incongruence, they are commonly diagnosed with a mental disorder and may be treated with gender reparative therapies. Acceptability in health care and public health services thus requires the depathologization of trans* identities and the enactment of legal protections against harmful gender reparative therapies and other “treatments” grounded in stigma. While these practices are increasingly understood to be inappropriate, unethical, and harmful to the goal of affirming gender identity and providing acceptable care, they have yet to be eliminated globally.^72^

Finally, progressively improving the quality of health care and underlying determinants of health for trans* populations will require more nuanced and increased research on all dimensions of trans* health. States must devote resources towards improving the understanding of health care and public health for trans* people. This should include funding high-quality epidemiological and clinical research on conditions that impact the health of trans* people, ensuring the health workforce is trained to understand and respond to trans* health needs, and ensuring quality standards and accreditation schemes for providers include thoughtful protocols that improve health services for trans* patients.

In meeting these attributes, governments must respect, protect, and fulfill the right to health. Respecting the right to trans* health requires the state itself to refrain from actions that deny or restrict access to health programs and services based on gender identity, including, for example, laws and policies that refuse to recognize a person’s gender identity and limit access to medical interventions and social services based on a person’s gender identity or gender expression. Laws and policies that permit — or in some cases actively encourage and entrench — discrimination against trans* persons must be replaced by laws and policies that prohibit discrimination on the basis of gender identity or gender expression. The enactment of anti-discrimination provisions, coupled with the enforcement of these provisions, comprise a key element in a state’s obligation to protect the right to health by preventing discrimination toward trans* populations across all sectors and communities. Likewise, states must affirmatively advance efforts in multiple areas to fulfill the right to trans* health through, among other things, funding supportive health programs and centering the health needs of trans* persons in national and regional health policies.

### B. Rights-Based Approaches to Trans* Health

Looking beyond the right to health, the “rights-based approach to health” provides tools to assist governments, health care practitioners, and communities in developing and implementing health policies, initiatives, and practices to promote the health and human rights of trans* people. This “rights-based approach” leverages cross-cutting human rights principles to guide and implement health activities through a participatory, inclusive, transparent, and responsive process. Rights-based approaches, with a growing evidence base showing their efficacy in improving health outcomes, can shape how states respond to their obligation to ensure that the benefits of public health measures are shared.^73^ It requires decision-makers to incorporate in their health actions core principles of: equality and non-discrimination (reflecting the indivisibility and universality of human rights and the dignity of all people), participation (especially of marginalized groups, like trans* people), and accountability.^74^

The principles of equality and non-discrimination require that all forms of discrimination in the realization of the human right to health be prohibited, prevented, and eliminated — and that states take affirmative measures to prioritize the most marginalized individuals who are likely to face the largest barriers to realizing their rights.^75^ In past and present day, trans* individuals suffer significant harm to their health as a result of systematic exposure to overlapping inequalities, including the legal, institutional, and social stressors associated with belonging to a marginalized group.^76^ Widespread misinformation, stigma, and discrimination also impact the health-seeking behavior of trans* individuals and the standard of care they receive in health care settings. Health is additionally influenced by workplace discrimination (forcing some people, notably trans women, into a narrow range of occupations that includes coercive or risky sex work) leading to under- or unemployment, increased exposure to harassment and abuse (often at the hands of law enforcement or healthcare providers themselves), and the constant threat of non-lethal and lethal violence.^77^ These dynamics — sometimes deliberately worsened by state and political actors — create meaningful obstacles to the progressive realization of health. Exacerbated by social and sexual network-level risks, these “situated vulnerabilities” interact to deprive trans* people of respect, opportunities, and dignity, and contribute to depression, anxiety, self-harm, and suicidal behavior.^78^ States must deliberately and affirmatively take steps to deconstruct these systems of discrimination, oppression, and structural inequity and support efforts to prioritize the health needs of trans* individuals.

Where trans* people have active, free, and meaningful opportunities to participate in all aspects of health-related decision-making, they are likely to experience health improvements.^79^ Trans* communities must be able to engage in the design, implementation, and monitoring of health research and interventions that concern them. To ensure meaningful participation, rights-based policies and practices must legitimize community representation and demonstrate government commitment to community involvement in health policy, especially from trans* civil society groups and multisectoral partnerships that link health with advocacy, social justice, and rights discourse.^80^ Meaningful participation by trans* people not only generates better understanding and effectively introduces and applies knowledge from those most affected by the policies and interventions being considered, but it can also educate and heighten awareness of trans* health considerations among people outside these communities, resulting in decreased stigma and marginalization.

Where trans* people have active, free, and meaningful opportunities to participate in all aspects of health-related decision-making, they are likely to experience health improvements. Trans* communities must be able to engage in the design, implementation, and monitoring of health research and interventions that concern them. To ensure meaningful participation, rights-based policies and practices must legitimize community representation and demonstrate government commitment to community involvement in health policy, especially from trans* civil society groups and multisectoral partnerships that link health with advocacy, social justice, and rights discourse.

To facilitate government accountability for the progressive realization of rights, health initiatives must empower rights-holders to seek redress from duty-bearers, imposing consequences for deficiencies. A variety of approaches and institutions might appropriately serve this function, including the use of human rights impact assessments of health policies and programs that affect trans* health and underlying determinants of health, increased health-related advocacy, monitoring and review structures, and enforcement through judicial systems.^81^ Assessing compliance with human rights obligations to trans* persons through national human rights institutions and international treaty bodies (such as the Committee on Economic, Social and Cultural Rights) provides a critical means to facilitate accountability through monitoring.^82^ These monitoring and review bodies have the capacity to exert high-level pressure on states to uphold and realize trans* rights and develop valuable recommendations that can drive future changes to policy and practice.

Given the longstanding neglect of these human rights norms and principles in developing health policies, programs, and practices in service of trans* people, there is an important opportunity to strengthen how the right to health and rights-based approach to health can be harnessed to advance health-related human rights. Trans* scholars and advocates do not universally accept the utility of human rights to facilitate improvements in health for trans* people. Critics have long feared that human rights discourses and obligations elevate ontological categories like sex, which serves to legitimize colonial, capitalist, and racialized systems and identity categories and therefore harms trans* social movements.^83^ Yet, human rights offer a universal legal foundation to advance the health of trans* people.^84^ To advance the health-related human rights of trans* under international law, the human rights framework must be reconceptualized — transcending the gender binary under international law in order to uphold the health-related human rights of trans* populations.

## IV. Moving Beyond the Gender Binary

As momentum both for and against the rights of trans* persons continues to grow, the COVID-19 pandemic — a new and substantial threat to the health and well-being of trans* communities — has taken hold around the world, exacerbating the discrimination, stigma, and violence faced by members across the entire LGBTQI+ community as economic stability, rising rates of violence, and enhanced criminalization compounds upon existing inequities within the community.^85^ As growing concern for setbacks in trans* progress continues to shape the political and legal landscape, a dynamic new report from the Independent Expert on SOGI—released in 2021 at the 47th UN Human Rights Council — helps articulate a pathway forward on how international human rights law can transcend the confines of the gender binary. Within the two-part SOGI publication named “Reports on Gender: The Law of Inclusion & Practices of Exclusion,” the document investigates both the long history of how gender has been interpreted within international human rights law and jurisprudence, as well as the rising anti-gender movement that has proved resistant to the incorporation of a gender-expansive framework under international human rights law.

One of the most important aspects of the publication is that it supports the understanding of international human rights law in a way that recognizes that gender is not reduced to the narrow limits of the binary, or one’s sex assigned at birth. This is first demonstrated by the research carried out by the Independent Expert, which shows that restrictive approaches to gender within human rights law are neither necessary nor legally or scientifically justified, and that fears of challenging them are senseless. In fact, in strong contrast to the rhetoric of anti-gender proponents at the UN, evidence within the report demonstrates that dismantling the social orthopedics of the gender binary across international law is necessary and urgent. This point was emphasized in the July 2022 report from the UN Special Rapporteur on the Right to the Highest Attainable Standard of Health, whose analysis highlights the physical, mental, and emotional violence experienced by people based on real or perceived sexual orientation, gender identity, gender expression and sex characteristics. In doing so, the Special Rapporteur underlines the critical necessity of adopting a non-binary approach to both gender and gender-based violence under international law, arguing that a failure to do so results in the continued institutionalization of violence against trans* populations under international law.^86^

As gender is a concept enshrined throughout international human rights law, its earliest uses can be traced back to 1993 in the Vienna Declaration and Programme of Action, as noted above. Although the initial interpretation of gender within the text was used to represent the experiences of cisgender women, the text allows for an unrestrictive interpretation. In gendered societies around the world, it is understood that *all people* are affected by “socio-cultural constructions that assign roles, behaviors, forms of expression, activities, and attributes according to the meaning given to sexual characteristics.”^87^ To put it simply, the gender order reaches us all, and the idea that only trans* individuals have a gender identity in need of protection under international human rights law is a false assumption. Rather, oppressive norms or practices that work to constrain individuals to certain gendered presentations and experiences is neither natural nor necessary but is instead sustained by a strong prescriptive vocation that aims to align certain physical characteristics with specific ways of living, including gender identity and expression.

At its heart, this is why principles of gender identity and expression are protected under international human rights law, and explicit references can be found to this in the work of the United Nations High Commissioner for Human Rights, the United Nations human rights mechanisms, as well as in regional human rights bodies.^88^ Additionally, this is why addressing violence and discrimination in both theoretical studies and policymaking requires the use of an analytical framework like that of intersectionality, which is capable of interpreting complex social hierarchies that cannot be captured by one-dimensional analyses or binary frameworks. The analytical power of this theoretical tool facilitates sophisticated examinations of the relationships in which different axes of social hierarchization (such as age, nationality, sexual orientation, gender identity, class, among others) are intertwined in complex ways in our societies and promote various layers of vulnerability and protection within the international legal framework.^89^

Through the use of the multidimensional lens of intersectionality, one is able to address diversity within all identity categories. In relation to gender in the international human rights context, this means that it can both challenge the narrow limits of the binary, as well as inscribe the understanding of gender relations in a web of multiple vectors of subjection.^90^ In practice, this makes it possible to “illuminate the actors, institutions, policies, and norms that intertwine to create a given situation” and sheds light on phenomena that would otherwise remain invisible, particularly in relation to the needs of the most marginalized communities.^91^ Prime examples of this include, the experiences of trans* and intersex children, whose age and gender identity intersect in a way that increases vulnerability to violations of their bodily integrity, in ways that are separate and unique from the experiences of both children and trans* and intersex adults. Or of trans* migrants, “who face discrimination both from destination country communities and from other migrants, so that even finding a safe space in which to meet becomes a barrier.”^92^

Despite the benefits that a more nuanced approach to gender could provide, efforts to transcend the gender binary in international human rights law have encountered strong backlash around the world. Such negative reactions are rhetorically asserted on the premise that recognizing gender as a socio-cultural construct, rather than an immutable condition predetermined by sex characteristics, has negative effects for groups and cultural institutions that should be protected. For some supporters of anti-gender ideology, what is at risk are traditional family values, the nation, and/or religious institutions; while others assert that questioning of the gender binary order threatens different aspects of the lives of cisgender women and girls (in particular) and children (in general). Nevertheless, the 2021 report by the Independent Expert effectively demonstrates that concerns regarding these alleged threats to the hard-fought human rights of cisgender women, girls, and children are not grounded in evidence. This is quite conspicuous in that, while some claims lack evidence, others present “evidence” that is either inconclusive or has been refuted by the scientific community. For example, the assertion that the participation of trans women and girls in sports will discourage the participation of cisgender women and girls, lacks foundation. In fact, there is empirical evidence to support the opposite.^93^

Most important to note from the report is that it is the backlash against trans* rights, and not the proposed adoption of a gender-expansive legal framework, that poses a significant risk to the rights of women and LGBTQI+ people. To the former because, the anti-gender movement contributing to the backlash utilizes a framework of biological determinism that ultimately undermines the autonomy and bodily integrity of all cisgender women, stripping them of their agency and reducing their role in society to their sex characteristics. And to the latter, because the backlash against LGBTQI+ people sets the stage for significant rights violations. These rights violations can play out in numerous detrimental ways for LGBTQI+ individuals, including: through the impact of stigma and discrimination on societal perceptions of LGBTQI+ identities (causing communities to perceive LGBTQI+ identities as immoral, criminal, and worthy of incarceration); restrictions on family life (as witnessed in legal restrictions on the right to same-sex marriage); restrictions on the legal recognition of gender identity (leaving individuals vulnerable to negative health outcomes, and lacking in social and political protections); and negative impacts on bodily integrity (resulting in substantial human rights abuses, such as the egregious practice of “corrective” rapes against lesbians, coercive anal examinations on gay men, the promotion of “conversion therapy” and unnecessary surgeries on intersex babies).

With the above crucial understanding that the reconceptualization of gender under international human rights law stands to positively transform the health of not only trans* communities, but cisgender women and girls, and broader LGBTQI+ communities as well, the global community has a tremendous opportunity at hand to address long-standing inequities and injustice through the adoption of a more inclusionary legal framework. As pressures rise across the international stage from advocates promoting SOGI-inclusive reforms, anti-gender actors working to prevent and reverse SOGI protections, and the collective multilateral network working to build coalitions across this issue to accelerate progress towards the health-related Sustainable Development Goals (SDGs), it is clear that conversations centered on transcending the confines of the gender binary will play a prominent role in the larger global health agenda in the coming years. Though the work to incorporate non-binary approaches to gender within global health frameworks is a mission that extends far beyond the incoming targets of the SDGs, and other critical initiatives like Universal Health Coverage (UHC) and Family Planning 2030 (FP2030), those nearing targets serve as a critical benchmark, whose success will not be achieved if a more authentic, intersectional, and inclusive framing of Gender Equality is not prioritized.

As the world sits on this precipice of a potentially transformative shift in the international human rights framework, the next spotlight moment in accelerating these conversations is already on the horizon. With a forthcoming report from the UN Independent Expert on SOGI rights scheduled to be delivered at the 50th session of the Human Rights Council, the topic on the agenda is the right to health for individuals with diverse sexual orientations and gender identities in the context of the Sustainable Development Goals.^94^ With the deadline for the 2030 Agenda rapidly approaching, including achievement of SDG 3: the right to good health and well-being for all, the global community has much to gain from working together to untangle international human rights law from the constraints of biological determinism — allowing the health-related rights of trans* and additional populations to flourish by moving beyond the limitations of an outdated binary system.

## Note

The authors have no conflicts to disclose.

## References

[1] M. Lugones , “The Coloniality of Gender,” in The Palgrave Handbook of Gender and Development, W. Harcourt (eds) (London: Palgrave Macmillan, 2016), *available at* < 10.1007/978-1-137-38273-3_2> (last visited July 12 2022); Z. Matebeni, S. Monro, and V. Reddy, (Eds.), *Queer in Africa: LGBTQI Identities, Citizenship, and Activism* (1st ed.) (Routledge, 2018), *available at* < 10.1007/978-1-137-38273-3_2> (last visited July 13, 2022).

[2] K. Steinmetz , “The OED Added the Word ‘Trans*.’ Here’s What It Means,” *Time*, April 3, 2018, *available at* <https://time.com/5211799/what-does-trans-asterisk-star-mean-dictionary/> (last visited July 13, 2022).

[3] S. L. Reisner et al., “Global Health Burden and Needs of Transgender Populations: A Review,” The Lancet 388, no. 10042 (2016): 412–436. 10.1016/S0140-6736(16)00684-X PMC7035595 27323919

[4] “Trans Rights Map 2022 Reveals Slow Comeback of Progress on Trans Rights,” TGEU, May 12, 2022, *available at* <https://tgeu.org/trans-rights-map-2022/> (last visited July 13, 2022).

[5] The United Nations. Universal Declaration of Human Rights, Preamble. 1948.

[6] The United Nations. Universal Declaration of Human Rights, Art. 2. 1948.

[7] UN General Assembly Res. 22/2263, Declaration on the Elimination of Discrimination Against Women. A/RES/22/2263, November 7, 1967, *available at* <undocs.org/A/RES/22/2263> (last visited July 13, 2022).

[8] International Convention on the Elimination of All Forms of Discrimination Against Women, G.A. Res. 24/180, Art. 3 (1979), *available at* <http://www.un.org/womenwatch/daw/cedaw/text/econvention.htm> (last visited July 13, 2022).

[9] S. Stryker , Transgender History: The Roots of Today’s Revolution (Seal Press, 2017).

[10] *Id*.; J. M. Jaque and J. Sánchez, “La Revolución De Marcia Alejandra,” La Tercera, April 13, 2018, *available at* <https://www.latercera.com/tendencias/noticia/la-revolucion-marcia-alejandra/132019/> (last visited July 13, 2022).

[11] J. Butler , *“*Bodies that Matter: On the Discursive Limits of “Sex*”* (New York: Routledge, 1993).

[12] C. Bunc and R. Carillo , “Women’s Rights are Human Rights: A Concept in the Making,” in Women and Girls Rising Progress and Resistance around the World, ed. E. Chessler and T. McGovern (New York: Routledge, 2015).

[13] UN General Assembly, Vienna Declaration and Programme of Action, A/CONF.157/23, Article 41 (1993), available from undocs.org/A/CONF.157/23.

[14] United Nations, International Conference on Population and Development: Programme of Action, A/CONF.171/13, Paragraph 7.3. (Cairo, Egypt, September 5-13, 1994).

[15] United Nations, Beijing Declaration and Platform of Action, adopted at the Fourth World Conference on Women, Article 95. (27 October 1995), *available at* <http://www.un.org/womenwatch/daw/beijing/pdf/BDPfA%20E.pdf> (last visited July 15, 2022).

[16] C. Garcia-Morena , “Reproductive Health and Justice: International Women’s Conference for Cairo ’94.” (New York: International Women’s Health Coalition and Citizenship, Studies, Information and Action, 1994).

[17] T. McGovern and A. Ahmed , “Equity in Health: Sexual and Reproductive Health and Rights” in *Foundations of Global Health & Human Rights* (2020).

[18] C. Balzer and C. LaGata , “Human Rights,” TSQ: Transgender Studies Quarterly 1, no. 1-2 (2014): 99–103, *available at* < 10.1215/23289252-2399731> (last visited July 15, 2022).

[19] R. Robles , T. Real , and G.M. Reed , “Depathologizing Sexual Orientation and Transgender Identities in Psychiatric Classifications,” Consortium Psychiatricum 2, no. 2 (2021): 45–53, doi:10.17816/CP61. PMC11272317 39070736

[20] S. D. Cochran , J. Drescher , E. Kismödi , A. Giami , C. García-Moreno , E. Atalla , A. Marais , E. Meloni Vieira , and G. M. Reed , “Proposed Declassification of Disease Categories Related to Sexual Orientation in The International Statistical Classification of Diseases and Related Health Problems (ICD-11),” Bulletin of the World Health Organization 92 (2014): 672–679, *available at* < 10.2471/BLT.14.135541> (last visited September 19, 2022). 25378758 PMC4208576

[21] M. E. Castro-Peraza , J. M. García-Acosta , N. Delgado , A. M. Perdomo-Hernández , M. I. Sosa-Alvarez , R. Llabrés-Solé , and N. D. Lorenzo-Rocha , “Gender Identity: The Human Right of Depathologization,” International Journal of Environmental Research and Public Health 16, no. 6 (2019): 978, *available at* < 10.3390/ijerph16060978> (last visited July 15, 2022) PMC6466167 30889934

[22] H. Feola , “It’s Time to Stop Gatekeeping Medical Transition,” *American Scientist*, February 18, 2022, *available at* <https://www.americanscientist.org/blog/macroscope/its-time-to-stop-gatekeeping-medical-transition> (last visited July 15, 2022).

[23] S.L. Schulz , “The Informed Consent Model of Transgender Care: An Alternative to the Diagnosis of Gender Dysphoria,” Journal of Humanistic Psychology 58, no. 1 (2017): 72–92, *available at* < 10.1177/0022167817745217> (last visited July 15, 2022).

[24] A. Suess Schwend , “Trans Health Care from a Depathologization and Human Rights Perspective,” Public Health Reviews 41, no. 1 (2020), *available at* < 10.1186/s40985-020-0118-y> (last visited July 15, 2022); K. Sheherezade, “Gender is not an Illness. How Pathologizing Trans People Violates International Human Rights Law,” *GATE* (2017), *available at* < https://gate.ngo/gender-is-not-an-illness/> (last visited September 19, 2022). PMC7031999 32099728

[25] M. Alipour , “Islamic Shari’a Law, Neotraditionalist Muslim Scholars and Transgender Sex-Reassignment Surgery: A Case Study of Ayatollah Khomeini’s and Sheikh Al-Tantawi’s Fatwas,” International Journal of Transgenderism 18, no. 1 (2016): 91–103, *available at* < 10.1080/15532739.2016.1250239> (last visited July 15, 2022).

[26] Balzer and LaGata, *supra* note 18.

[27] C. G. Cabrera , “Argentina Recognizes Non-Binary Identities: Decree Allows for Third Gender Option in Identification Documents,” Human Rights Watch, July 22, 2021, *available at* <https://www.hrw.org/news/2021/07/22/argentina-recognizes-non-binary-identities> (last visited July 15, 2022).

[28] J. Meritus , “Applying the Gatekeeper Model of Human Rights Activism: The U.S. Based Movement for LGBT Rights” in The International Struggle for New Human Rights, ed. C. Bob (2009): 52–67.

[29] From E. Jourdaan , “The Challenge of Adopting Sexual Orientation Resolutions at the UN Human Rights Council,” Journal of Human Rights Practice 8 (2016): 298–310.

[30] D. McGoldrick , “The Development and Status of Sexual Orientation Discrimination under International Human Rights Law,” Human Rights Law Review 16 (2016): 613–668.

[31] D. Karsay , “How Far has SOGI Advocacy come at the UN and where is it Heading? Assessing Sexual Orientation, Gender Identity, and Intersex Activism and Key Developments at the UN 2003-2014,” ARC International (2014).

[32] P. Hunt , “The right of everyone to the enjoyment of the highest attainable standard of physical and mental health” (E/CN.4/2004/49) (United Nations, 2004).

[33] M.-A. George , “The LGBT Disconnect: Politics and Perils of Legal Movement Formation” Wisconsin Law Review 504 **(**2018): 504-591.

[34] *Id*.

[35] *Id*.

[36] F. Girard, “United Nations: Negotiation Sexual Rights and Sexual Orientation at the UN,” in Sex Politics: From the Frontlines, ed. R. Parker , R. Petchesky and R. Sember (Sexuality Policy Watch, 2004), *available at* <http://www.sxpolitics.org/frontlines/book/index.php> (last visited July 18, 2022).

[37] International Commission of Jurists (ICJ), *Yogyakarta Principles - Principles on the Application of International Human Rights Law in Relation to Sexual Orientation and Gender Identity*, March 2007, *available at* <https://data.unaids.org/pub/manual/2007/070517_yogyakarta_principles_en.pdf> (last visited July 18, 2022).

[38] *Id*.

[39] D. Sanders , “International: The Role of the Yogyakarta Principles,” OutRight Action International, August 4, 2008, *available at* <https://outrightinternational.org/content/international-role-yogyakarta-principles> (last visited July 18, 2022).

[40] E. Jourdaan , “The Challenge of Adopting Sexual Orientation Resolutions at the UN Human Rights Council,” Journal of Human Rights Practice 8 (2016): 298–310.

[41] UN Office of the High Commissioner for Human Rights (OHCHR), “Resolutions on Sexual Orientation, Gender Identity and Sex Characteristics: OHCHR and the Human Rights of LGBTI People,” *available at* <https://www.ohchr.org/en/sexual-orientation-and-gender-identity/resolutions-sexual-orientation-gender-identity-and-sex-characteristics> (last visited July 18, 2022).

[42] UN Human Rights Council, Human rights, sexual orientation and gender identity: resolution adopted by the Human Rights Council, (June 17, 2011). A/HRC/RES/17/19.

[43] Arc International, “United Nations Human Rights Chief Publishes Groundbreaking Report on Sexual Orientation and Gender Identity,” ARC International, December 15, 2011, *available at* <https://arc-international.net/united-nations-human-rights-chief-publishes-ground-breaking-report-on-sexual-orientation-and-gender-identity/> (last visited July 18, 2022).

[44] UN Human Rights Council, Human rights, sexual orientation and gender identity: resolution adopted by the Human Rights Council, (2 October 2014). A/HRC/RES/27/32, *available at* <http://ap.ohchr.org/documents/dpage_e.aspx?si=A/HRC/RES/27/32> (last visited July 18, 2022).

[45] OHCHR, “Vitit Muntarbhorn, Former Independent Expert,” OHCHR, 2021, *available at* <https://www.ohchr.org/en/issues/sexualorientationgender/pages/vititmuntarbhorn.aspx> (last visited July 18, 2022).

[46] “The Yogyakarta Principles Plus 10,” Yogyakarta Principles, November 10, 2017, *available at* <https://yogyakartaprinciples.org/wp-content/uploads/2017/11/A5_yogyakartaWEB-2.pdf> (last visited July 18, 2022).

[47] V. Madrigal-Borloz , “Reports on Gender: The Law of Inclusion & Practices of Exclusion,” OHCHR, July 2021, *available at* <https://www.ohchr.org/Documents/Issues/SexualOrientation/IESOGI/Reports_on_Gender_Final_Summary.pdf> (last visited July 18, 2022).

[48] L. Armitage, “Explaining Backlash to Trans and Non-Binary Genders in the Context of UK Gender Recognition Act Reform,” INSEP - Journal of the International Network for Sexual Ethics & Politics 8, no. Special Issue 2020 (2020): 11–35, *available at* < 10.3224/insep.si2020.02> (last visited July 18, 2022).

[49] R. Sanders, “Norm Spoiling: Undermining the International Women’s Rights Agenda,” International Affairs 94, no. 2 (2018): 271–291.

[50] A.M. Goetz , “The New Competition in Multilateral Norm-Setting: Transnational Feminists & the Illiberal Backlash,” Daedalus 149, no. 1 (2020): 160–179, *available at* < 10.1162/daed_a_01780> (last visited July 18, 2022).

[51] *Id*.

[52] K. Burns , “The Rise of Anti-Trans ‘Radical’ Feminists, Explained,” *Vox*, September 5, 2019, *available at* <https://www.vox.com/identities/2019/9/5/20840101/terfs-radical-feminists-gender-critical> (last visited July 18, 2022).

[53] WDI, “Declaration on Women’s Sex-Based Rights,” Women’s Declaration International, 2022, *available at* <https://www.womensdeclaration.com/en/> (last visited July 18, 2022).

[54] J. Holzman, “The Unlikely Political Alliance Against Trans Care,” *POLITICO*, March 8, 2022, *available at* <https://www.politico.com/newsletters/the-recast/2022/03/08/politics-transgender-health-care-feminists-religious-conservatives-00015307> (last visited July 18, 2022).

[55] Armitage, *supra* note 48.

[56] S. Murphy and L. Brooks , “UK Government Drops Gender Self-Identification Plan for Trans People,” *The Guardian*, September 22, 2020, *available at* <https://www.theguardian.com/society/2020/sep/22/uk-government-drops-gender-self-identification-plan-for-trans-people> (last visited July 18, 2022).

[57] Global Philanthropy Project (GPP), “Meet the Moment: A Call for Progressive Philanthropic Response to the Anti-Gender Movement,” Global Philanthropy Project, July 28, 2021, *available at* <https://globalphilanthropyproject.org/2020/11/12/meet-the-moment/> (last visited July 18, 2022).

[58] V. Madrigal-Borloz , G. Misra , P. Grotenhuis , M. C. Grinspan , A. Rueda , and R. Alsalem , “LGBTI and Feminist Movements: Pushing back on the Pushback,” Webinar from Outright Action International. November 17, 2021, *available at* <https://www.youtube.com/watch?v=bGAukvzYidk> (last visited July 18, 2022).

[59] UN Committee on Economic, Social and Cultural Rights (CESCR), at paragraph 8.

[60] UN Committee on Economic, Social and Cultural Rights (CESCR), *supra* note 59, at paragraph 12(a).

[61] UN Committee on Economic, Social and Cultural Rights (CESCR), *supra* note 59, at paragraph 12(b).

[62] J. M. Koch , C. McLachlan , C. J. Victor , J. Westcott , and C. Yager, “The Cost of Being Transgender: Where Socio-Economic Status, Global Health Care Systems, and Gender Identity Intersect,” Psychology & Sexuality 11, no. 1-2 (2020): 103–119.

[63] S. Winter , M. Diamond , J. Green , D. Karasic , T. Reed, S. Whittle , and K. Wylie , “Transgender People: Health at the Margins of Society,” The Lancet 388, no. 10042 (2016): 390–400. 10.1016/S0140-6736(16)00683-8 27323925

[64] L. G. Arnull , A. Kapilashrami , and M. Sampson , “Visualizing Patterns and Gaps in Transgender Sexual and Reproductive Health: A Bibliometric and Content Analysis of Literature (1990-2020),” International Journal of Transgender Health, November 30, 2021. 10.1080/26895269.2021.1997691 PMC10601526 37901061

[65] S. Winter , E. Settle , K. Wylie , S. Reisner , M. Cabral , G. Knudson , and S. Baral , “Synergies in Health and Human Rights: A Call to Action to Improve Transgender Health,” The Lancet 388, no. 10042 (2016): 318–321. 10.1016/S0140-6736(16)30653-5 PMC6098969 27323921

[66] S. Winter, “Lost in Transition: Transpeople, Transprejudice and Pathology in Asia,” The International Journal of Human Rights 13, no. 2-3 (2009): 365–390.

[67] G. Rodriguez-Ferrand , “Argentina: New Law on Transgender Rights Approved,” The Library of Congress, May 16, 2012, *available at* <https://www.loc.gov/item/global-legal-monitor/2012-05-16/argentina-new-law-on-transgender-rights-approved/> (last visited July 18, 2022).

[68] D. Operario and T. Nemoto , “On Being Transnational and Transgender: Human Rights and Public Health Considerations,” American Journal of Public Health 107, no. 10 (2017): 1537–1538. 28902560 10.2105/AJPH.2017.304030 PMC5607703

[69] K. Kosenko , L. Rintamaki , S. Raney , and K. Maness, “Transgender Patient Perceptions of Stigma in Health Care Contexts,” Medical Care (2013): 819–822. 23929399 10.1097/MLR.0b013e31829fa90d

[70] D. M. Warner and Arunab Harish Mehta . “Identifying and addressing barriers to transgender healthcare: where we are and what we need to do about it.” Journal of General Internal Medicine 36, no. 11 (2021): 3559–3561. 34254221 10.1007/s11606-021-07001-2 PMC8606364

[71] J. Drescher , P. Cohen-Kettenis , and S. Winter , “Minding the Body: Situating Gender Identity Diagnoses in the ICD-11,” International Review of Psychiatry 24, no. 6 (2012): 568–577. 23244612 10.3109/09540261.2012.741575

[72] F. Bustreo , P. Hunt , and World Health Organization, Women’s and Children’s Health: Evidence of Impact of Human Rights, World Health Organization, 2013.

[73] F. Bustreo , and C.F.J. Doebbler , “The Rights-Based Approach to Health,” Foundations of Global Health & Human Rights (2020): 89.

[74] UN Committee on Economic, Social and Cultural Rights (CESCR), General Comment No. 14: The Right to the Highest Attainable Standard of Health (Art. 12 of the Covenant), August 11,2000, E/C.12/2000/4.

[75] J. C. Phelan , B. G. Link , and P. Tehranifar , “Social Conditions as Fundamental Causes of Health Inequalities: Theory, Evidence, and Policy Implications,” Journal of Health and Social Behavior 51, no. 1_suppl (2010): S28–S40. 20943581 10.1177/0022146510383498

[76] J. M. Grant, L.A. Mottet, J. Tanis , J. Harrison , J.L. Herman , and M. Keisling , *Injustice at Every Turn. A Report of the National Transgender Discrimination Survey* (2011).

[77] S. Winter , E. Settle , K. Wylie , S. Reisner , M. Cabral , G. Knudson , and S. Baral , “Synergies in Health and Human Rights: A Call to Action to Improve Transgender Health,” The Lancet 388, no. 10042 (2016): 318–321. 10.1016/S0140-6736(16)30653-5 PMC6098969 27323921

[78] UN Committee on Economic, Social and Cultural Rights (CESCR), *supra* note 59, at paragraph 17.

[79] B.M. Meier , C. Pardue , and L. London , “Implementing Community Participation through Legislative Reform: A Study of the Policy Framework for Community Participation in the Western Cape Province of South Africa,” BMC International Health and Human Rights 12, no. 1(2012): 1–14. 22920557 10.1186/1472-698X-12-15 PMC3532148

[80] F. Bustreo and C. F.J. Doebbler, “The Rights-Based Approach to Health,” *Foundations of Global Health & Human Rights* (2020): 89.

[81] B.M. Meier , H.E. Huffstetler , and J. Bueno de Mesquita , “Monitoring and Review to Assess Human Rights Implementation,” *Foundations of Global Health & Human Rights* (2020).

[82] D. Irving , “Against the Grain: Teaching Transgender Human Rights,” Sexualities 16, no. 3-4 (2013): 319–335.

[83] B. Ramirez Guzmán, “Colonialidad y Cis-Normatividad,” *Iberoamérica Social*, January 13, 2015, *available at* <https://iberoamericasocial.com/colonialidad-y-cis-normatividad-conversando-con-viviane-vergueiro/> (last visited July 18, 2022); V. Vergueiro, “Viviane Vergueiro: Despatologizar é descolonizar,” GATE: Global Action for Trans Equality, March 27, 2020, *available at* <https://gate.ngo/es/viviane-vergueiro-despatologizar-es-descolonizar/> (last visited July 18, 2022); T. Powell, S. Shapiro, and E. Stein, “Transgender Rights as Human Rights,” *AMA Journal of Ethics* 18, no. 11 (2016): 1126-1131. 10.1001/journalofethics.2016.18.11.pfor3-1611 27883304

[84] European Region International Lesbian, Gay, Bisexual, Trans and Intersex Association, (ILGA Europe), “COVID-19 and Specific Impact on LGBTI People and What Authorities Should be Doing to Mitigate Impact,” June 2020, *available at* <https://www.ilga-europe.org/report/covid-19-and-specific-impact-on-lgbti-people-and-what-authorities-should-be-doing-to-mitigate-impact/> (last visited September 19, 2022).

[85] UN Special Rapporteur on the Right to the Highest Attainable Standard of Health, “Report of the Special Rapporteur on the Right of Everyone to the Enjoyment of the Highest Attainable Standard of Physical and Mental Health, Tlaleng Mofokeng,” July 2022, *available at* <https://www.ohchr.org/en/documents/thematic-reports/ahrc5028-violence-and-its-impact-right-health-report-special-rapporteur> (last visited July 18, 2022).

[86] International Lesbian, Gay, Bisexual, Trans and Intersex Association (IGLA World), “SOGIESC Opportunities in the 44th Human Rights Council Session 30 June - 20 July 2020,” August 2020, *available at* <https://ilga.org/downloads/HRC44_SOGIESC_session_report.pdf> (last visited July 18, 2022).

[87] Human Rights Council resolutions 17/19 and 27/32; A/HRC/29/23, paras. 21, 78 and 79; A/HRC/29/33/Add.1, paras. 86, 88 and 111 (q); General Assembly resolution 69/182; Committee on Economic, Social and Cultural Rights, general comment No. 20 (2009), para. 27; Committee on Economic, Social and Cultural Rights, general comment No. 22 (2016), paras. 23 and 40; Committee on the Rights of the Child, general comment No. 15 (2013), para. 8; Committee on the Rights of the Child, general comment No. 20 (2016), paras. 33 and 34; Committee against Torture, general comment No. 2 (2007), para. 21; CCPR/C/KWT/CO/3, paras. 12 and 13; and CCPR/C/RUS/CO/7, para. 10.

[88] K. Crenshaw , “Demarginalizing the Intersection of Race and Sex: A Black Feminist Critique of Antidiscrimination Doctrine, Feminist Theory and Antiracist Politics,” University of Chicago Legal Forum 1989, no. 1 (1989), *available at* <http://chicagounbound.uchicago.edu/uclf/vol1989/iss1/8> (last visited July 19, 2022).

[89] M. Pérez , “Interseccionalidad,” in Nuevo Diccionario de Estudios de Género y Feminismos, ed. S. Gamba and T. Diz (Buenos Aires: Biblos, 2021).

[90] *AWID Intersectionality: A Tool for Intersectionality: A Tool for Gender and Economic Justice Gender and Economic Justice. Women’s Rights and Economic Change* No. 9. (2004), *available at* <https://www.awid.org/sites/default/files/atoms/files/intersectionality_a_tool_for_gender_and_economic_justice.pdf> (last visited July 19, 2022).

[91] ILGA World, *supra* note 86.

[92] S.K. Goldberg , *Fair Play: The Importance of Sports Participation for Transgender Youth*, February 8, 2021, *available at* <https://www.americanprogress.org/article/fair-play/> (last visited July 19, 2022).

[93] OHCHR, *Call for Inputs: Report to the Human Rights Council on the Realisation of the Right of Persons Affected by Violence and Discrimination Based on Sexual Orientation and Gender Identity to the Enjoyment of the Highest Attainable Standard of Physical and Mental Health, in Relation to SDG3* (2021), *available at* <https://www.ohchr.org/EN/Issues/SexualOrientationGender/Pages/CFI-IE-SOGI-report-50thsession-HRC.aspx> (last visited July 19, 2022).

